# Hydrostatic Pressure Regulates Oxidative Stress through microRNA in Human Osteoarthritic Chondrocytes

**DOI:** 10.3390/ijms21103653

**Published:** 2020-05-21

**Authors:** Sara Cheleschi, Marcella Barbarino, Ines Gallo, Sara Tenti, Maria Bottaro, Elena Frati, Stefano Giannotti, Antonella Fioravanti

**Affiliations:** 1 Department of Medicine, Surgery and Neuroscience, Rheumatology Unit, Azienda Ospedaliera Universitaria Senese, Policlinico Le Scotte, 53100 Siena, Italy; ins.gll3@gmail.com (I.G.); sara_tenti@hotmail.it (S.T.); elena.frati@unisi.it (E.F.); fioravanti7@virgilio.it (A.F.); 2 Department of Medical Biotechnologies, University of Siena, 53100 Siena, Italy; marcella.barbarino@unisi.it (M.B.); mariaeusebia.bottaro@gmail.com (M.B.); 3 Sbarro Institute for Cancer Research and Molecular Medicine, Center for Biotechnology, College of Science and Technology, Temple University, Philadelphia, PA 19122, USA; 4 Department of Medicine, Surgery and Neurosciences, Section of Orthopedics and Traumatology, University of Siena, Policlinico Le Scotte, 53100 Siena, Italy; stefano.giannotti@unisi.it

**Keywords:** hydrostatic pressure, microRNA, oxidative stress, chondrocytes, osteoarthritis, Wnt/β-catenin, mechanical loading, miR-34a, miR-146a, miR-181a

## Abstract

Hydrostatic pressure (HP) modulates chondrocytes metabolism, however, its ability to regulate oxidative stress and microRNAs (miRNA) has not been clarified. The aim of this study was to investigate the role of miR-34a, miR-146a, and miR-181a as possible mediators of HP effects on oxidative stress in human osteoarthritis (OA) chondrocytes. Chondrocytes were exposed to cyclic low HP (1–5 MPa) and continuous static HP (10 MPa) for 3~h. Metalloproteinases (MMPs), disintegrin and metalloproteinase with thrombospondin motif (ADAMTS)-5, type II collagen (Col2a1), miR-34a, miR-146a, miR-181a, antioxidant enzymes, and B-cell lymphoma 2 (BCL2) were evaluated by quantitative real-time polymerase chain reaction qRT-PCR, apoptosis and reactive oxygen species ROS production by cytometry, and β-catenin by immunofluorescence. The relationship among HP, the studied miRNA, and oxidative stress was assessed by transfection with miRNA specific inhibitors. Low cyclical HP significantly reduced apoptosis, the gene expression of *MMP-13*, *ADAMTS5*, miRNA, the production of superoxide anion, and mRNA levels of antioxidant enzymes. Conversely, an increased *Col2a1* and *BCL2* genes was observed. β-catenin protein expression was reduced in cells exposed to HP 1–5 MPa. Opposite results were obtained following continuous static HP application. Finally, miRNA silencing enhanced low HP and suppressed continuous HP-induced effects. Our data suggest miRNA as one of the mechanisms by which HP regulates chondrocyte metabolism and oxidative stress, via Wnt/β-catenin pathway.

## 1. Introduction

Articular cartilage is constantly exposed to a wide range of static and dynamic cycles of loading that can vary from 0.7 to 20 MPa depending on body weight, posture, and physical activity [1,2,3]. Moderate loads are safe and contribute to maintain cartilage homeostasis and integrity, while excessive or static stresses could be deleterious, inducing its degradation, and contributing to the onset and the progression of osteoarthritis (OA) [4,5]. At the cellular level, a dynamic physiological compression promotes chondrocyte proliferation and activity, whereas injurious static stress induces opposing, detrimental effects on cell anabolism reducing the synthesis of extracellular matrix (ECM) components [6,7,8]. A number of in vitro studies reported the role of hydrostatic pressure (HP) as modulators of morphology and metabolism of chondrocytes; the effects of HP depended of magnitude, duration, and frequency of loading [7,9,10,11,12,13]. Interestingly, preliminary experiments on human OA chondrocyte cultures demonstrated the ability of HP to modify the expressional levels of some microRNAs (miRNA), involved in the pathogenesis of OA, and to regulate oxidative stress balance [14,15,16]. In particular, it has been observed that cycles of low physiological HP (1–5 MPa) decreased the expression levels of *miR-181a* [17], a post-transcriptional regulator of pro-inflammatory processes and cartilage degradation during OA [18]. A mechano-responsiveness of *miR-146a* was firstly identified after a mechanical injuring pressure of 10 MPa and following cycles of sinusoidal low HP [15,16,19,20].

Growing evidence demonstrates that an excessive production of reactive oxygen species (ROS) and a reduction of antioxidant factors contribute to cartilage degradation, subchondral bone changes, and synovial inflammation occurring in OA joints. The imbalance between oxidant/antioxidant system inhibits the synthesis of ECM, cell migration, activates matrix degrading enzymes production and apoptosis, leading to a loss of cartilage integrity [21]. Furthermore, ROS overproduction participates to exacerbate synovitis and to release catabolic cytokines such as interleukin (IL)-1β and tumor necrosis factor alfa (TNF)-α; on the other hand, inflamed synovial cells stimulate the synthesis of newly ROS, creating a vicious circle [22,23]. Mechanical load seems to be effective in the modulation of oxidant/antioxidant system even if the current data available from the literature are scarce and controversial [13,24,25,26].

Lately, several in vitro researches on human OA chondrocyte cultures highlight a cross talk between miRNA and oxidative stress. Interestingly, it has been demonstrated that some specific miRNA, identified as oxidative stress-responsive factors [27], are modulated by ROS which can induce or suppress miRNA expression and contribute to downstream biological function through regulation of target genes [28]. In addition, miRNA may influence the production of free radicals and the expression of the components of cellular antioxidant machinery [29,30].

The purpose of the present study aimed at investigating the role of *miR-34a*, *miR-146a*, and *miR-181a* as possible mediators of HP regulation of oxidative stress balance in human OA chondrocyte exposed to cycles of low sinusoidal HP (1–5 MPa) and static continuous HP (10 MPa), for a period of 3~h. In particular, under these experimental conditions, we analyzed the gene expression of matrix degrading enzymes, metalloproteinases *(MMP)-13*, disintegrin and metalloproteinase with thrombospondin motif *(ADAMTS)-5*, and collagen type 2 (*Col2a1*), as well as the ratio of apoptosis and the mRNA levels of the anti-apoptotic mediator B-cell lymphoma 2 *(BCL)2*. The mitochondrial ROS production and the transcriptional levels of antioxidant enzymes, superoxide dismutase *(SOD)-2* and nuclear factor erythroid 2 like 2 (*NRF2*), and of the studied miRNA were also evaluated. Then, to examine the relationship among HP, the studied miRNA and oxidative stress, the cultured cells were transiently transfected with miRNA specific inhibitors. Finally, the analysis of Wnt/β-catenin pathway in HP-induced effects was further assessed.

## 2. Results

### 2.1. Hydrostatic Pressure Regulates Chondrocyte Metabolism

**Figure 1:**
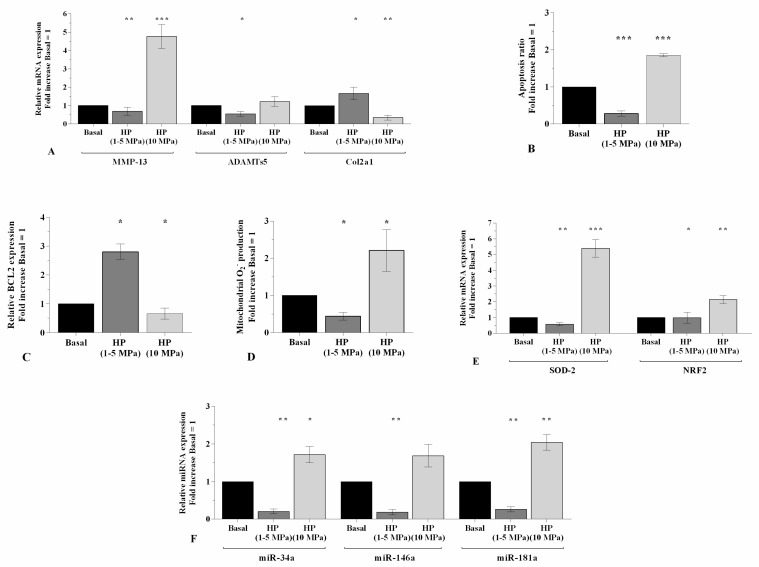
Effects of HP exposure on chondrocyte metabolism. (**A**,**C**,**E**,**F**) Expression levels of *MMP-13*, *ADAMTS-5*, *Col2a1*, *BCL2*, *SOD-2*, *NRF2*, *miR-34a*, *miR-146a*, *miR-181a* analyzed by quantitative real-time polymerase chain reaction PCR. (**B**) Apoptosis detection performed by flow cytometry analysis and measured with Annexin Alexa fluor 488 assay. Data were expressed as the percentage of positive cells for Annexin-V and propidium iodide (PI) staining. (**D**) Mitochondrial superoxide anion production evaluated by MitoSox Red staining at flow cytometry. Human OA chondrocytes were evaluated at basal condition and after 3~h of low sinusoidal (1–5 MPa) or static continuous (10 MPa) HP exposure. The gene expression, the ratio of apoptosis and the production of superoxide anion were referenced to the ratio of the value of interest and the value of basal condition, reported equal to 1. Data were expressed as mean ± standard deviation SD of triplicate values. * *p* < 0.05, ** *p* < 0.01, *** *p* < 0.001 versus basal condition.

Figure 1 shows the effects of 3~h-application of low sinusoidal or static continuous HP cycles in regulating the gene expression of matrix degrading enzymes, *Col2a1*, and miRNA, the percentage of apoptotic cells and the oxidative stress balance, in OA chondrocyte cultures. The exposure of the cells to low sinusoidal HP (1–5 MPa) determined a significant reduction of *MMP-13* (*p* < 0.01), *ADAMTS-5* (*p* < 0.05), and an up-regulation of *Col2a1* mRNA levels (*p* < 0.05), in comparison to basal condition (Figure 1A). A decrease of apoptotic cells (*p* < 0.001, Figure 1B) and an increase of *BCL2* gene (*p* < 0.05, Figure 1C) were also found. Furthermore, low HP reduced mitochondrial superoxide anion production (*p* < 0.05, Figure 1D), *SOD-2* (*p* < 0.01) and *NRF2* (*p* < 0.05) (Figure 1E) transcriptional levels, and *miR-34a*, *miR-146a*, *miR-181a* (*p* < 0.01, Figure 1F) gene expression. On the contrary, a cycle of static continuous HP (10 MPa) significantly up-regulated the gene expression of *MMP-13* (*p* < 0.001), *SOD-2* (*p* < 0.001), *NRF2* (*p* < 0.01) of the studied *miRNA* (*p* < 0.01), and decreased the mRNA levels of *Col2a1* (*p* < 0.01) and *BCL2* (*p* < 0.05). This pressure significantly induced apoptosis and ROS production (*p* < 0.001, *p* < 0.05, respectively, Figure 1A–F).

### 2.2. MiRNA Specific Inhibitors Mediate HP Effect on miR-34a, miR-146a, and miR-181a Gene Expression

**Figure 2:**
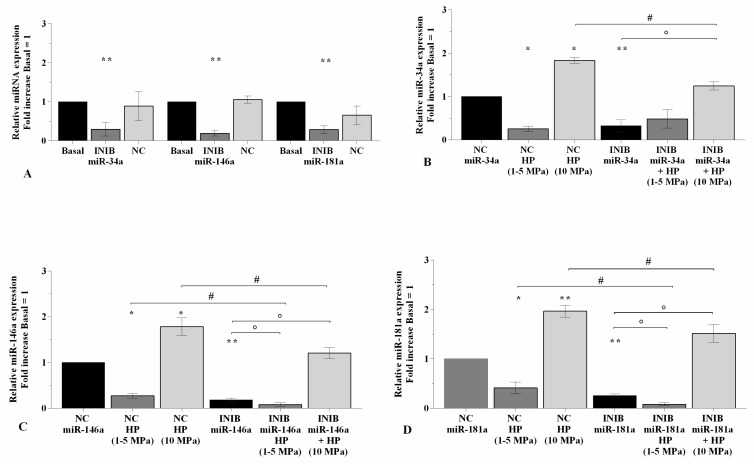
miRNA silencing limits the effect of HP on *miR-34a*, *miR-146a,* and *miR-181a*. (**A**–**D**) Expression levels of *miR-34a*, *miR-146a*, and *miR-181a* analyzed by real-time PCR. Human OA chondrocytes were evaluated at basal condition, after 24~h of transfection with miR-34a, miR-146a, and miR-181a inhibitors (50 nM) or NC (5 nM), and after 3~h of low sinusoidal (1–5 MPa) or static continuous (10 MPa) HP exposure. The gene expression was referenced to the ratio of the value of interest and the value of basal condition, reported equal to 1. Data were expressed as mean ± SD of triplicate values. * *p* < 0.05, ** *p* < 0.01 versus basal condition or NC. ° *p* < 0.05 versus inhibitor. # *p* < 0.05 versus HP. INIB = Inhibitor, NC = negative control siRNA.

To confirm the effect of HP in modulating *miR-34a*, *miR-146a*, and *miR-181a* expression, OA cells were transiently transfected with miRNA specific inhibitors for 24~h before the application 3~h of the studied cycles of pressurization (Figure 2). Real-time PCR analysis revealed the ability of inhibitors to significantly reduce the gene expression of *miR-34a*, *miR-146a*, and *miR-181a* (*p* < 0.01) in comparison to basal condition and negative control (NC) (Figure 2A). The exposure to low sinusoidal HP significantly down-regulated the transcriptional levels of the studied miRNA (*p* < 0.01, Figure 2B–D) in OA chondrocytes transfected with NC. Afterwards the silencing of miRNA, the cells subjected to low HP showed a reduction of *miR-146a* and *miR-181a* expression levels (*p* < 0.05) compared to the chondrocytes transfected with the inhibitors or exposed to low HP alone, while no significant modifications on *miR-34a* were observed (Figure 2B–D). Conversely, when NC transfected cells were exposed to static continuous HP a significant increase of miRNA expression was showed (*p* < 0.05 for *miR-34a*, *p* < 0.01 for *miR-146a*, *miR-181a*, Figure 2B–D). This up-regulation (*p* < 0.05) was also observed in inhibited chondrocytes put through to high HP with respect to the cell cultures incubated with miRNA inhibitors. However, miRNA gene expression in transfected cells exposed to pressurization was significantly lower (*p* < 0.05) than what is observed after continuous HP exposure alone (Figure 2B–D).

### 2.3. MiRNA Silencing Regulates Cartilage Turnover Altered by HP

**Figure 3:**
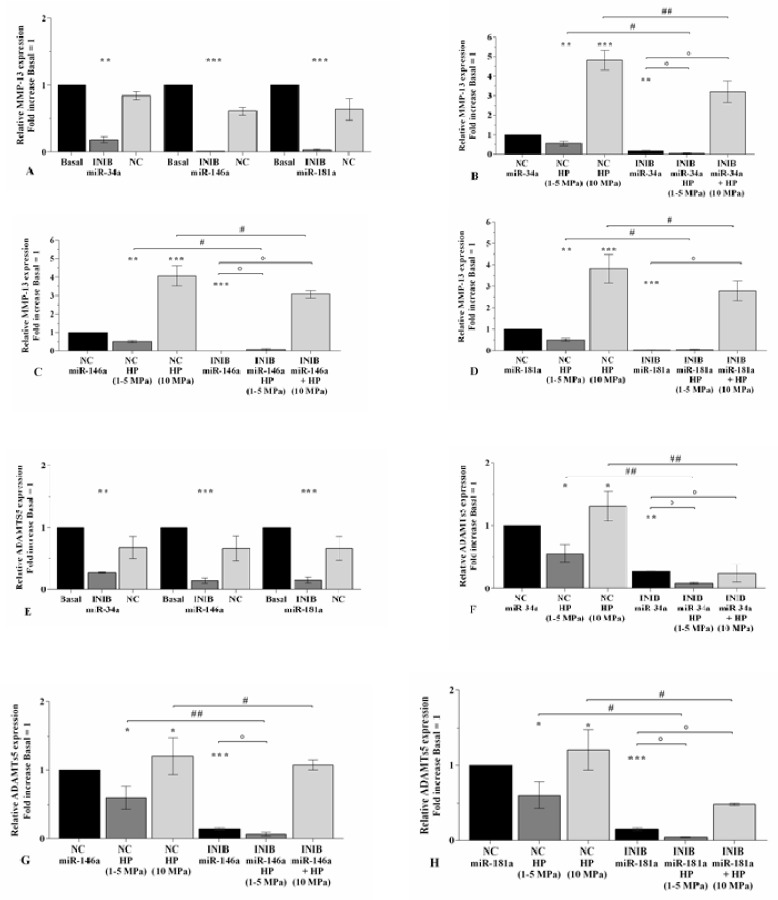
miRNA inhibition reduces the expression of matrix degrading enzymes altered by HP. (**A**–**H**) Expression levels of *MMP-13* and *ADAMTS-5* analyzed by real-time PCR. Human osteoarthritic (OA) chondrocytes were evaluated at basal condition, after 24~h of transfection with miR-34a, miR-146a, and miR-181a inhibitors (50 nM) or NC (5 nM), and after 3~h of low sinusoidal (1**–**5 MPa) or static continuous (10 MPa) HP exposure. The gene expression was referenced to the ratio of the value of interest and the value of basal condition or NC, reported equal to 1. Data were expressed as mean ± SD of triplicate values. * *p* < 0.05, ** *p* < 0.01, *** *p* < 0.001 versus basal condition or NC. ° *p* < 0.05 versus inhibitor. # *p* < 0.05, ## *p* < 0.01 versus HP. INIB = Inhibitor, NC = negative control siRNA.

**Figure 4:**
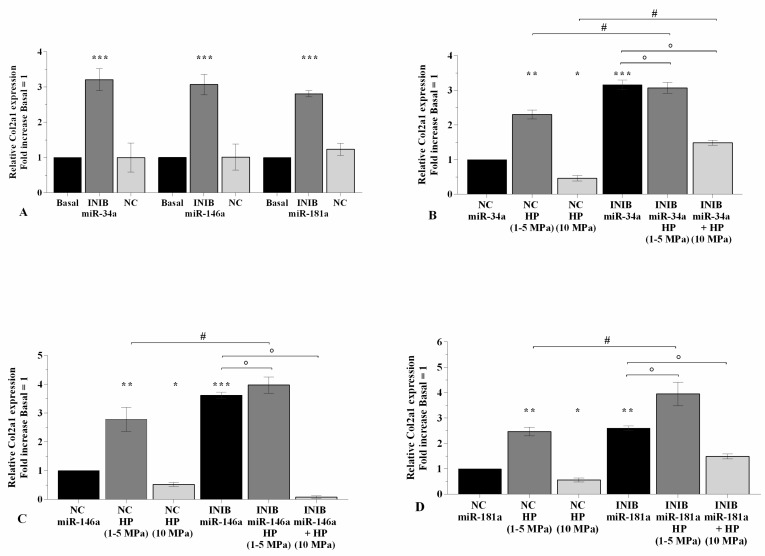
miRNA inhibition induces the expression of *Col2a1* altered by HP. (**A**–**D**) Expression levels of *Col2a1* analyzed by real-time PCR. Human OA chondrocytes were evaluated at basal condition, after 24~h of transfection with miR-34a, miR-146a, and miR-181a inhibitors (50 nM) or NC (5 nM), and after 3~h of low sinusoidal (1–5 MPa) or static continuous (10 MPa) HP exposure. The gene expression was referenced to the ratio of the value of interest and the value of basal condition or NC, reported equal to 1. Data were expressed as mean ± SD of triplicate values. * *p* < 0.05, ** *p* < 0.01, *** *p* < 0.001 versus basal condition or NC. ° *p* < 0.05 versus inhibitor. # *p* < 0.05 versus HP. INIB = Inhibitor, NC = negative control siRNA.

Figure 3 and Figure 4 summarize the modulation of cartilage metabolism, induced by 3~h of pressurization system, following transfection of OA chondrocytes with miRNA specific inhibitors for 24~h. The silencing of miR-34a, miR-146a, and miR-181a caused a significant down-regulation of *MMP-13* and *ADAMTS5*, and an increase of *Col2a1* expression levels (*p* < 0.01, *p* < 0.001) in comparison to basal condition and NC (Figure 3A,E, and Figure 4A). Low HP exposure significantly reduced the transcriptional levels of *MMP-13* (*p* < 0.01), *ADAMTS5* (*p* < 0.05), and up-regulated *Col2a1* mRNA (*p* < 0.01) in chondrocytes transfected with NC (Figure 3B–D, F–H, and Figure 4B–D). When the cells were silenced with miRNA inhibitors, a strongly significant reduction of *MMP-13* and *ADAMTS5* (*p* < 0.05), and an increase of *Col2a1* (*p* < 0.05) were observed in low HP-exposed cells in comparison to chondrocytes incubated with the only inhibitors or subjected to low HP alone (Figure 3B–D, F–H, and Figure 4B–D). No significant changes in *MMP-13* expression were observed in low HP-exposed cells after silencing of miR-181a with respect to the only inhibitor (Figure 3D). On the other hand, the effects of continuous HP on the expression levels of *MMP-13* and *ADAMTS5* were significantly counteracted by the transfection of the cells with miR-34a, miR-146a, and miR-181a inhibitors (Figure 3B–D, F–H). MiR-34a silencing significantly limited the reduction of *Col2a1* mRNA (*p* < 0.05) induced by 10 Mpa HP (Figure 4B), while no modifications were found after the transfection of miR-146a and miR-181a (Figure 4C,D).

### 2.4. MiRNA Inhibitors Mediate the Effect of HP on Chondrocyte Apoptosis

**Figure 5:**
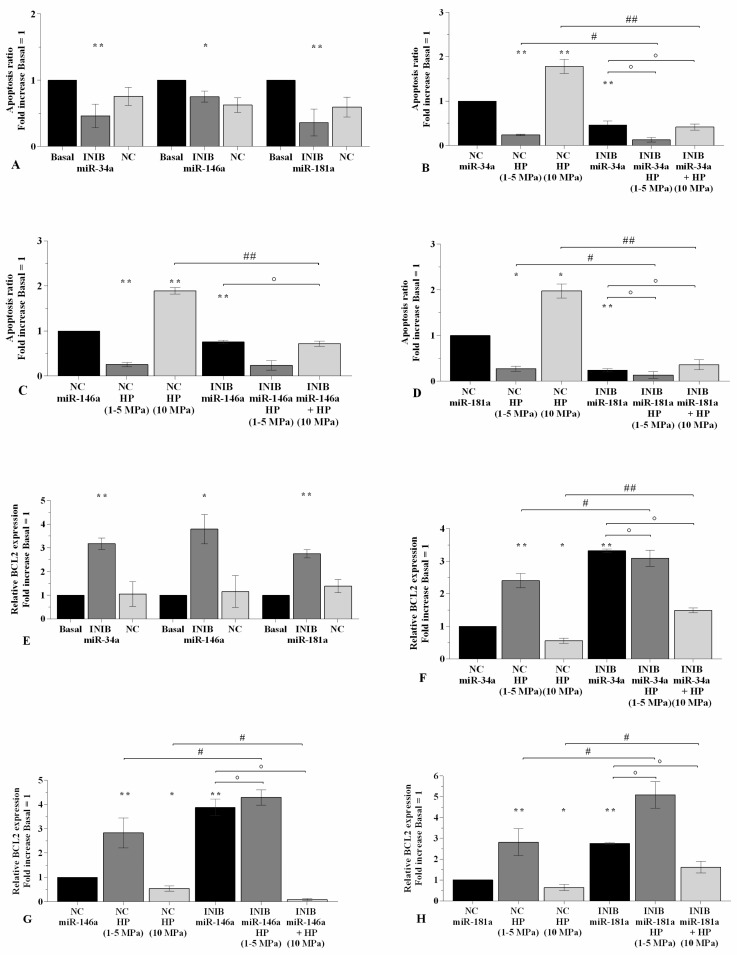
miRNA inhibition mediates HP effect on apoptosis. (**A**–**D**) Apoptosis detection performed by flow cytometry analysis and measured with Annexin Alexa fluor 488 assay. Data were expressed as the percentage of positive cells for Annexin-V and propidium iodide (PI) staining. (**E**–**H**) Expression levels of *BCL2* analyzed by real-time PCR. Human OA chondrocytes were evaluated at basal condition, after 24~h of transfection with miR-34a, miR-146a, and miR-181a inhibitors (50 nM) or NC (5 nM), and after 3~h of low sinusoidal (1**–**5 MPa) or static continuous (10 MPa) HP exposure. The ratio of apoptosis and the gene expression were referenced to the ratio of the value of interest and the value of basal condition or NC, reported equal to 1. Data were expressed as mean ± SD of triplicate values. * *p* < 0.05, ** *p* < 0.01 versus basal condition or NC. ° *p* < 0.05 versus inhibitor. # *p* < 0.05, ## *p* < 0.01 versus HP. INIB = Inhibitor, NC = negative control siRNA.

The regulation of cellular apoptosis was evaluated in OA chondrocytes subjected to 3~h-application of low sinusoidal or static continuous HP programs, after their transient transfection of 24~h with miR-34a, miR-146a, and miR-181a specific inhibitors (Figure S1, Figure 5). Flow cytometry analysis showed a significant decrease of apoptosis ratio (*p* < 0.05, *p* < 0.01, Figure 5A) and an up-regulation of *BCL2* gene expression (*p* < 0.05, *p* < 0.01, Figure 5E) in OA chondrocytes transfected with miR-34a, miR-146a, and miR-181a inhibitors when compared to basal condition and NC. The exposure to low HP reduced the amount of apoptotic cells (*p* < 0.01) and raised the mRNA levels of *BCL2* (*p* < 0.01) in NC transfected chondrocytes. Conversely, a cycle of continuous HP increased the apoptosis ratio (*p* < 0.01) with a concomitant down-regulation of *BCL2* gene levels (*p* < 0.05) (Figure 5B–D, F–H). The silencing of miR-34a and miR-181a significantly increased the effects of low HP on apoptosis and *BCL2* mRNA, while limited those induced by continuous HP, in comparison to what is observed by using the only inhibitors or the exposure to HP alone (Figure 5B,D,F,H). When the cells were transfected with miR-146a inhibitor, the effects of the studied pressurizations on apoptosis and *BCL2* expression where significantly strengthened when compared to the cells cultivated with the only inhibitor (Figure 5C,G). miR-146a inhibitor also reduced the apoptosis (*p* < 0.01) and increased *BCL2* gene (*p* < 0.05) caused by the exposure of OA chondrocytes to 10 Mpa HP with respect to continuous HP alone (Figure 5C,G). On the other hand, miR-146a silencing significantly enhanced the gene expression of *BCL2* induced by low HP (*p* < 0.05) in comparison to the effect of the only HP, while no changes were observed in terms of apoptosis ratio (Figure 5C,G).

### 2.5. The Silencing of miRNA Regulates Oxidative Stress Following HP Exposure

**Figure 6:**
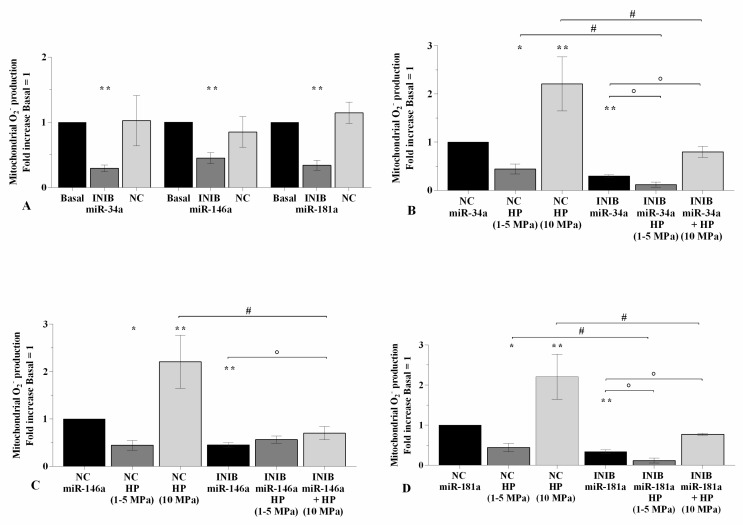
miRNA silencing regulates oxidative stress following HP exposure. (**A**–**D**) Mitochondrial superoxide anion production evaluated by MitoSox Red staining at flow cytometry. Human OA chondrocytes were evaluated at basal condition, after 24~h of transfection with miR-34a, miR-146a, and miR-181a inhibitors (50 nM) or NC (5 nM), and after 3~h of low sinusoidal (1–5 MPa) or static continuous (10 MPa) HP exposure. The production of superoxide anion was referenced to the ratio of the value of interest and the value of basal condition or NC, reported equal to 1. Data were expressed as mean ± SD of triplicate values. * *p* < 0.05, ** *p* < 0.01 versus basal condition or NC. ° *p* < 0.05 versus inhibitor. # *p* < 0.05 versus HP. INIB = Inhibitor, NC = negative control siRNA.

**Figure 7:**
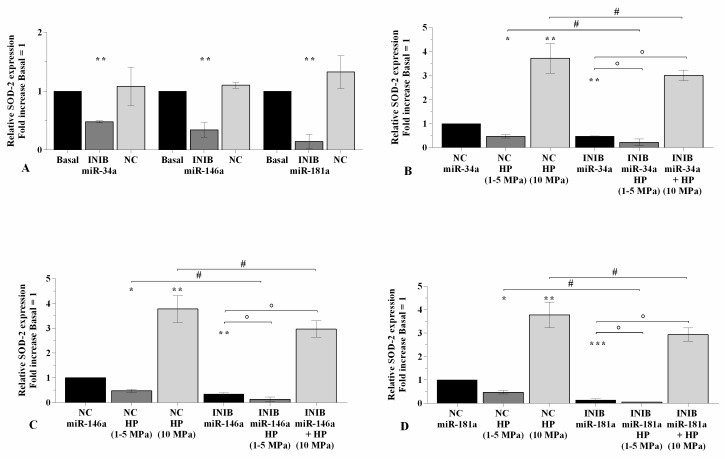
miRNA silencing regulates *SOD-2* expression following HP exposure. (**A**–**D**) Expression levels of *SOD-2* analyzed by real-time PCR. Human OA chondrocytes were evaluated at basal condition, after 24~h of transfection with miR-34a, miR-146a, and miR-181a inhibitors (50 nM) or NC (5 nM), and after 3~h of low sinusoidal (1–5 MPa) or static continuous (10 MPa) HP exposure. The gene expression was referenced to the ratio of the value of interest and the value of basal condition or NC, reported equal to 1. Data were expressed as mean ± SD of triplicate values. * *p* < 0.05, ** *p* < 0.01, *** *p* < 0.001 versus basal condition or NC. ° *p* < 0.05 versus inhibitor. # *p* < 0.05 versus HP. INIB = Inhibitor, NC = negative control siRNA.

**Figure 8:**
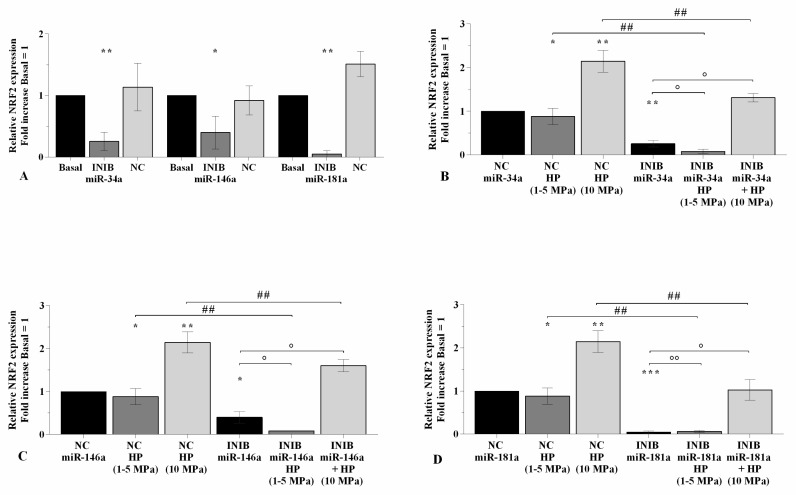
miRNA silencing regulates *NRF2* expression following HP exposure. (**A**–**D**) Expression levels of *NRF2* analyzed by real-time PCR. Human OA chondrocytes were evaluated at basal condition, after 24~h of transfection with miR-34a, miR-146a, and miR-181a inhibitors (50 nM) or NC (5 nM), and after 3~h of low sinusoidal (1–5 MPa) or static continuous (10 MPa) HP exposure. The gene expression was referenced to the ratio of the value of interest and the value of basal condition or NC, reported equal to 1. Data were expressed as mean ± SD of triplicate values. * *p* < 0.05, ** *p* < 0.01, *** *p* < 0.001 versus basal condition or NC. ° *p* < 0.05, °° *p* < 0.01 versus inhibitor. ## *p* < 0.01 versus HP. INIB = Inhibitor, NC = negative control siRNA.

In Figure 6, Figure 7, and Figure 8 is reported the regulation of oxidant/antioxidant balance observed in OA chondrocytes upon 24~h of transfection with miR-34a, miR-146a, and miR-181a inhibitors and 3~h of loading application (Figure S2). The use of specific miRNA inhibitors significantly limited the mitochondrial superoxide anion production (*p* < 0.01, Figure 6A), the expression levels of *SOD-2* (*p* < 0.01, Figure 7A), and *NRF2* (*p* < 0.01, *p* < 0.001, Figure 8A) in OA chondrocytes with respect to basal condition and NC. Low HP induced a significant reduction of ROS release (*p* < 0.05) and of anti-oxidant enzymes transcriptional levels (*p* < 0.05), while opposite results were obtained after the application of static continuous pressure, in NC transfected cells (*p* < 0.05, Figure 6B–D, Figure 7B–D, Figure 8B–D). The beneficial effect exerted by low HP on redox balance was significantly enhanced when OA chondrocytes were transfected with miRNA inhibitors in comparison to the cells incubated with the only inhibitors or HP (*p* < 0.05, *p* < 0.01, Figure 6B–D, Figure 7B–D, Figure 8B–D), except for the production of superoxide anion after inhibition of miR-146a.

On the contrary, miRNA silencing limited the increased superoxide anion levels and *SOD-2* and *NRF2* mRNA induced by 10 MPa pressurization, respectively to the effects of HP alone (*p* < 0.05, *p* < 0.01, Figure 6B–D, Figure 7B–D, Figure 8B–D).

### 2.6. MiRNA Silencing Regulates β-catenin Expression Altered by HP

**Figure 9:**
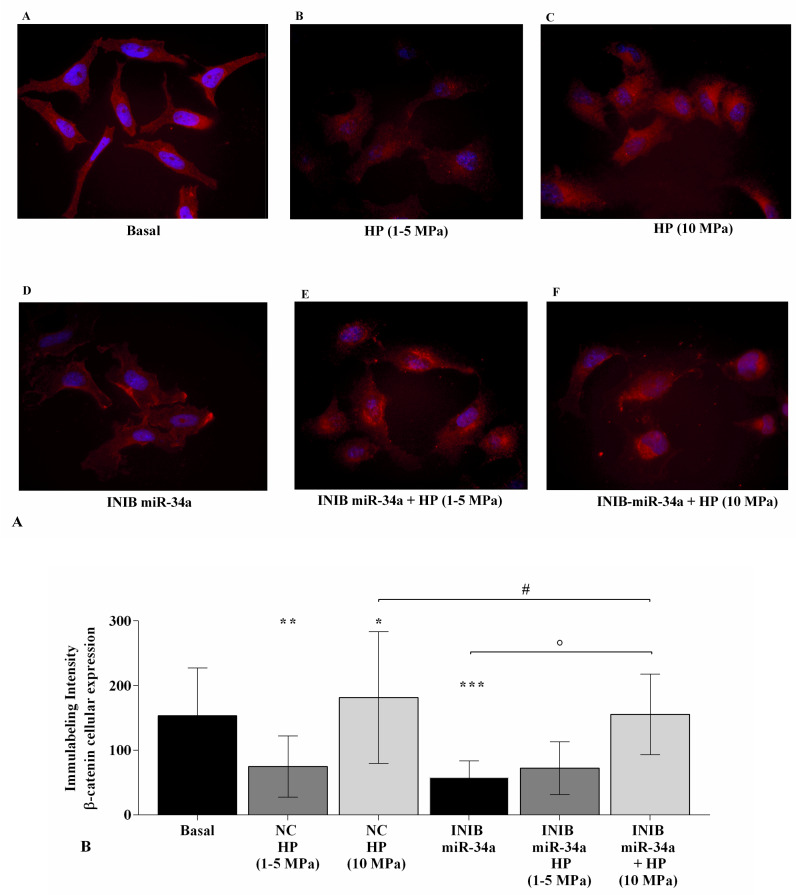
miR-34a inhibition mediates HP effect on β-catenin protein expression. Immunofluorescence labeling of β-catenin localization. Human OA chondrocytes were evaluated at basal condition, after 24~h of transfection with miR-34a inhibitor, and after 3~h of low sinusoidal (1–5 MPa) or static continuous (10 MPa) HP exposure. (**A**) Representative immunocytochemical images of the cells showing localization of β-catenin (red); nuclei were stained with DAPI (blue). Original magnification 400×. Scale bar: 20 µm. (**B**) The histogram of immunolabeling intensity was plotted for the nuclear and cytoplasm expression for β-catenin. Data were expressed as mean ± SD of triplicate values. * *p* < 0.05, ** *p* < 0.01, *** *p* < 0.001 versus basal condition. ° *p* < 0.05 versus inhibitor. # *p* < 0.05 versus HP. INIB = Inhibitor.

To evaluate the possible implication of Wnt/β-catenin pathway in the regulation of HP-mediated effects, OA chondrocytes were transiently transfected (24~h) with miR-34a inhibitor and then subjected to cycles of HP (3~h). Figure 9A,B shows the cytoplasmic and nuclear signal intensity of β-catenin, assessed at immunofluorescence analysis, in HP-exposed chondrocytes silenced with miR-34a inhibitor. At basal condition, β-catenin signal was low and mainly detected in the cytoplasm of the cells. β-catenin cytoplasmic and nuclear intensity appeared to be significantly reduced (*p* < 0.05) following 3~h of low HP exposure, while resulted increased (*p* < 0.05) in static continuous HP-exposed cells, in comparison to baseline. The transfection with miR-34a inhibitor significantly decreased β-catenin signal (*p* < 0.001) with a resulting lower immunolabeling intensity when compared with basal condition (Figure 9A,B); miRNA silencing slight enhanced, even if not significant, the positive effect of low HP in reducing β-catenin intensity (*p* < 0.05), whereas it significantly limited those of injured continuous HP (*p* < 0.05).

## 3. Discussion

Osteoarthritis is the most common form of chronic musculoskeletal disorder and the leading cause of pain and impairment in adult and elderly populations. Since the disease typically induces functional decline and reduction of quality of life, it represents, therefore, a massive world-wide healthcare and a financial burden, especially due to the increasing rates of obesity, sedentary lifestyle, and longevity [31,32]. The pathogenesis of the disease is complex and remains largely unknown, however, it is assumed that multiple factors such as genetics, aging, obesity, and biomechanical stimuli have an active role in cartilage degradation and synovial inflammation [31,32].

The effect of biomechanical stress on cartilage has not completely understood and depends on the intensity, duration, and frequency of loading, which regulate the metabolic activity of chondrocytes, by influencing normal balance between their anabolic and catabolic processes, and eventually induce OA [7,33]. Various biological systems have been implemented to apply mechanical stimuli to chondrogenic cells to mimic in vivo joint loading conditions. In the present study, we reported the results obtained using a prototype of HP system, for in vitro cultures, able to generate pressurization programs that can vary for magnitude, frequency, and duration. In particular, we applied 3~h of a cyclic sinusoidal low pressure of 1 to 5 MPa, to represent the range of loading encountered in human joints during a normal gait, or a static continuous HP of 10 MPa, exceeding the range of physiological loading measured *in vivo*, and appropriated to simulate a traumatic injury [7,34,35]. Under these experimental conditions, we evaluated the effects of HP on cartilage turnover, on apoptosis and oxidative stress balance, and on the modulation of some miRNA implicated in OA pathogenesis. Further, we hypothesized the role of miRNA and Wnt/β catenin signaling pathway in mediating HP effects on the studied cellular processes.

The destruction of articular cartilage represents one of main feature of OA. The structural breakdown of ECM components, including proteoglycans and Col2a1, is known to be the result of an increased catabolic activity of cartilage degrading enzymes, MMPs and ADAMTS [36]. Metalloprotease-13 is a major collagenase that targets cartilage for degradation. It not only targets Col2a1, one of the primary structural components of the cartilage ECM, in cartilage for degradation, but also degrades proteoglycan, types IV and type IX collagen, osteonectin and perlecan. It has been shown an up-regulation of *MMP-13* transcriptional levels in OA chondrocytes [15], and that *MMP-13*-overexpressing transgenic mice develop a spontaneous OA-like articular cartilage destruction phenotype [37]. The two main aggrecanases, ADAMTS-4 and -5, are known to mediate tissue damage, in particular, by targeting and degrading aggrecan; they are over-expressed in OA chondrocytes and synovial fibroblasts [15,38,39]. The effect of mechanical loading on cartilage biology has been amply studied, underling its essential role in modulating cartilage metabolism and homeostasis [7,12,40,41].

In the present study, cycles of low HP (1–5 MPa) applied to OA chondrocytes, induced a reduction of *MMP-13* and *ADAMTS-5* gene expression and an up-regulation of *Col2a1* mRNA levels; on the other hand, a continuous static loading of 10 MPa limited the expression of anabolic molecules in favor of catabolic processes. These results confirmed what is previously reported by other Authors. Hosseini et al. [42] demonstrated that the exposure of human mesenchymal stem cells at 3 MPa cyclic HP determined an increased gene expression of *Col2a1*; Cheleschi et al. [15] subjected human OA chondrocytes at cycles of sinusoidal HP of 1 to 5 MPa causing a reduction of matrix degrading enzymes. In addition, the exposure of human chondrocytes to mechanical overloading of 10 or 24 MPa induced the expression of breakdown enzymes and a reduction of matrix components [7,43,44].

Apoptosis is a physiological process of programmed cell death implicated in maintaining homeostasis of articular cartilage. Previous studies reported that chondrocytes apoptosis can occur in response to mechanical injury in vitro [19,45,46]. These findings proved an increased cell death via apoptosis when human cartilage explants were subjected to a single static mechanical stress of 14 MPa for 500 ms under radially unconfined compression [46], or when OA chondrocytes were pressurized with 4.5 MPa or 10 MPa of loading for different timing protocols [19,45]. More recently, Li et al. [47] examined rabbit mandibular condylar chondrocytes after static pressures of 100 kPa and 200 kPa for 3~h showing an increased chondrocyte proliferation at 100 kPa and decreased at 200 kPa with an induction of apoptosis. Similarly, in our study we observed an increased percentage of apoptotic cells after the exposure of OA chondrocytes at 3~h of static continuous HP of 10 MPa. Furthermore, we firstly demonstrated a reduction of cell death when a low sinusoidal HP of 1 to 5 MPa was applied, with a concomitant increased expression of the anti-apoptotic marker *BCL2*. Our results appear in contrast with the data obtained by Beecher et al. in 2007 [48]; the Authors exposed human cylindrical cartilage explants to radially unconfined cyclic axial compression (3600 cycles, 1 Hz, 50% duty cycle), using two physiologic loads (2 MPa and 5 MPa, 24~h), founding 30% of chondrocytes died in the superficial zone within 24~h of exposure to 5 MPa axial compression. The apparent discrepancy with our data is probably related to the difference in the experimental procedure performed, and, in particular, in the samples employed (chondrocytes vs. cartilage tissue) and the pressurization system applied.

These results demonstrate the relevance of mechanical loading as possible regulator of chondrocyte metabolism. A moderate stress keeps cartilage integrity, while a mechanical overloading contributes to induce cartilage degradation in terms of activation of catabolic processes, and thereby amplify apoptosis and oxidative stress signaling, thus reducing the number of functional chondrocytes [41].

Reactive oxygen species are normally produced at low levels on articular chondrocytes and act as signaling intermediates in multiple pathways participating to modulate ECM synthesis and breakdown, cytokines and growth factors production, apoptosis and gene expression. Under physiological conditions, ROS accumulation is controlled by an endogenous antioxidant defense system. In OA, the redox balance has lost and the excessive ROS formation participates to alter gene expression and promotes genomic instability, exacerbating cartilage destruction, synovitis, and apoptosis [21,22,23]. It has been reported an association between mechanical stress and the regulation of oxidant/antioxidant system, even if, data from the literature are poor [13,24,25,26]. Among them, Young et al. [25] observed an increased production of ROS, as hydrogen peroxide and hydroxyl radicals, after applying 24~h of static compression ranged from 40 to 120 psi at porcine chondrocytes. A similar increase of ROS species was observed by Rieder et al. [13] upon the application, at human adipose tissue derived stromal cells, of 4~h of intermittent stimulation of 0 and 4 bars for 5 consecutive days/week for 21 days. The results of our study partially sustain these data even though the experimental protocols are not comparable. We demonstrated an excessive mitochondrial ROS production following the exposure of OA chondrocytes to continuous HP of 10 MPa, while a positive reduction in cells subjected to cyclic low HP of 5 MPa was observed. However, our results revealed that the overproduction of ROS was associated to the increased expression levels of antioxidant factors, *SOD-2* and *NRF2*, maybe as a radical scavenging mechanism activated by the cells against the excessive amount of ROS [29,30,49]. Indeed, since the deleterious effects of abundant ROS sources, the antioxidant system, driven by *NRF2* transcription factor, react to deactivate ROS and protect the cells from oxidative damage. When activated, *NRF2* is released from its repressive cytosolic protein Kelch-like ECH associated protein1 (KEAP1), translocates into the nucleus, and stimulates the expression of cytoprotective genes encoding antioxidant enzymes, such as *SOD-2* and *catalase*, for neutralizing ROS species [21,29,30,49,50].

These findings suggest how a specific application and intensity of HP stimulation could differently influence the oxidative stress balance and contribute to regulate ROS-induced damage on chondrocytes and ECM components. 

Recently, the importance of mechanical loading or HP as modulator of miRNA gene expression has been amply reported [16,51,52]. miR-181a is known to be associated to inflammatory processes and cartilage degradation [18]; furthermore, it resulted highly expressed in OA chondrocytes and decreased when the cells were subjected to cycles of low physiological HP (1–5 MPa) [17]. miR-34a has been classified as an anti-proliferative factor regulating cell cycle arrest or senescence [53]. Its abnormal expression is correlated with several human diseases and its active role in the development and in the progression of OA has been demonstrated [53,54]. The role of miR-146a in OA has not still completely elucidated. Some Authors showed its up-regulation in OA cartilage with a low grade on the Mankin scale, while miRNA expression decreased with the progression of the disease [55]. On the other hand, the down-regulation of *miR-146a* in OA chondrocytes in comparison to healthy cells has been reported [15]. A mechano-responsive activity of this miRNA was firstly described by Jin et al. [19] observing that a mechanical injuring pressure of 10 MPa induced the up-regulation of *miR-146a* and a simultaneous activation of apoptosis in human normal chondrocytes. Later, another group of Investigators [15] found its expression significantly increased following cycles of low sinusoidal HP (1–5 MPa). The results of the present study confirm the current literature showing a reduced expression of *miR-146a* and *miR-181a* in OA chondrocytes exposed to 1 to 5 MPa, while their up-regulation is observed when an overloading HP of 10 MPa was applied. To the best of our knowledge, we firstly report the responsiveness of miR-34a at different HP applications; we have observed the down-regulation of miRNA expression following a cycle of low HP, and an increase of its gene levels in 10 MPa HP-exposed cells. Taken together, this evidence demonstrates the effect of HP in inducing significant changes in miRNA expression in human chondrocytes, suggesting miRNA as potential mediators by which mechanical loading regulates chondrocytes metabolism. 

At this regard, a number of studies have identified some miRNA, including miR-34a, miR-146a, and miR-181a, as regulators of oxidative stress machinery and apoptosis signaling cascade. Transfection experiments on human primary OA chondrocytes and synoviocytes showed the direct targeting of miR-34a, miR-146a, and miR-181a on SIRT-1/p53, Smad4, and BCL2 signaling pathways responsible for the regulation of cell proliferation and apoptosis in OA damage [19,29,30,54]. In addition, the modulation of Smad4 induced by miR-146a in OA chondrocytes apoptosis and ECM degradation is influenced by the application of an injuring pressure of 10 MPa [19]. Our results are consistent with these data, indeed we observed a reduction of chondrocytes apoptosis and an increased expression of *BCL2* genes by silencing miR-34a, miR-146a, and miR-181a, as well as a decreased catabolic activity of *MMP-13* and *ADAMTS-5*. Furthermore, the inhibition of miRNA counteracted the negative effects of the overloading HP on these factors and enhanced those of a low cyclic pressure. Additionally, it has been reported that the transient transfection of miR-34a, miR-146a, and miR-181a down-regulated the expression of antioxidant enzymes with a simultaneous decrease of mitochondrial intracellular ROS levels in PBMC of Holstein cows, in HUVEC lines, and in primary OA chondrocytes and synoviocytes [29,30,56,57]. According to this evidence, in this study, the silencing of miR-34a and miR-181a reduced the production of mitochondrial superoxide anion and the mRNA levels of *SOD-2* and *NRF2* in OA chondrocytes, increasing the activity of low cyclic HP and limiting the effects of static continuous loading. The production of superoxide anion, which was reduced after the application of low cyclic HP, seemed not to be conditioned by the inhibition of miR-146a; this missed result can be probably due to the variability usually observed in in vitro experiments, since the majority of the data, including those related to the modulation of antioxidant enzymes, confirmed the ability of miR-146a to influence the effect of low HP.

This is the first report showing the involvement of miRNA as possible mediator of HP effects on oxidative stress balance probably related to their direct effect on NRF2 activity. Therefore, it has been demonstrated the implication of miR-34a and miR-146a in modulating *NRF2* gene levels and NRF2-dependent antioxidant signaling [58,59]. However, our data reported a reduction of *SOD-2* and *NRF2* transcriptional levels when the miRNA were inhibited. We can hypothesize that this finding could be due to the direct binding of miR-34a and miR-181a on *SIRT-1* mRNA, decreasing the activity of this gene, known as regulator of the expression of several antioxidant genes [19,29,30,54]. Thus, a condition of oxidative stress reduced both the protein and mRNA levels of *SIRT-1*, whilst up-regulating the expression of *miR-34a* and *miR-181a*.

Articular cartilage growth and homeostasis are modulated by the activation of several pathways including the canonical β-catenin-dependent Wnt signaling pathway [60,61]. The activation of Wnt signaling, by different stimuli, causes nuclear β-catenin translocation and initiates the transcription of many genes responsible for bone and cartilage turnover [60,61]. Protein levels of Wnt and β-catenin are increased in human OA chondrocytes; this abnormal signaling activation determines the up-regulation of *MMPs* and *ADAMTS*, the reduction of *Col2a1*, proteoglycans, and aggrecans expression, and induces apoptosis signaling, contributing to articular cartilage damage [15,62,63,64,65,66]. Furthermore, it has been demonstrated the activation of Wnt/β-catenin pathway in articular cartilage of injured exercise-induced OA rat model [67], as well as the regulation of *β-catenin* mRNA and protein expression in in vitro chondrocytes subjected to intermittent cyclic mechanical tension and low HP [15,64,68,69]. In particular, Niu et al. [64] exposed chondrogenic-differentiated ATDC5 cells to a 12% cycle tension load for 1, 2, 4, or 8~h, and reported increased β-catenin protein expression initially after tension, then decreased over the mechanical loading period. Similarly, rat primary chondrocytes were subjected to cyclic mechanical strain with a 0.5 Hz sinusoidal curve at 10% elongation for 8~h a day, and an induction of β-catenin levels was observed [68]. Cheleschi et al. [15] demonstrated a reduction of β-catenin protein expression in OA chondrocytes exposed to 3~h of low sinusoidal HP (1–5 MPa), while no consistent load-dependent changes in total or active β-catenin-levels were observed by Praxenthaler et al. [69] after the application of a single dynamic compression-episode. In the present study, we partially confirm these results observing an increased β-catenin protein expression in OA chondrocytes upon application of a static continuous HP of 10 MPa, while its activation was limited in physiological HP-exposed cells (1–5 MPa). We further hypothesize that the effect of mechanical loading or, specifically, of HP on Wnt/β-catenin pathway could be mediated by miRNA. Indeed, several investigations demonstrated, in human and rat OA chondrocyte cultures, the direct targeting of some miRNA, implicated in OA damage, on Wnt or β-catenin proteins [70,71,72,73]. Likewise, our data reported a reduced β-catenin protein expression when OA chondrocytes were transiently silenced with miR-34a specific inhibitor; moreover, miRNA inhibition limited the negative effects of overloading HP on β-catenin expression and enhanced, even if not significantly, those of low cyclic pressure. Further experiments are required to better investigate this aspect.

The preliminary results of our study show the effects of HP as regulator of chondrocyte metabolism, apoptosis signaling, and oxidative stress balance; however, we need to take into consideration that there are a number of different systems to apply mechanical loading or HP in vitro that make difficult to do any comparisons with results available from the literature.

## 4. Materials and Methods

### 4.1. Samples Collection and Cell Cultures

Human OA articular cartilage was obtained from femoral heads of five non-obese (BMI ranging from 20 to 23 kg/m2) and non-diabetic patients (two men and three women, age ranging from 65 to 75) with coxarthrosis according to ACR criteria [74], subjected to total hip arthroplasty. OA grades ranged from moderate to severe, and cartilage showed typical OA changes, as the presence of chondrocyte clusters, fibrillation, and loss of metachromasia (Mankin degree 3–7) [75]. OA chondrocytes derived from the area adjacent to the OA lesion. The femoral heads were supplied by the Orthopaedic Surgery, University of Siena, Italy. The use of human articular samples was permitted after the authorization of the Ethic Committee of Azienda Ospedaliera Universitaria Senese/Siena University Hospital (decision no. 13931/18), and informed consent of the donor. Chondrocytes were isolated immediately after surgery. In brief, cartilage fragments were aseptically dissected from each donor and processed by an enzymatic digestion with trypsin (Sigma-Aldrich, Milan, Italy) for 15 min at 37 °C and then with type IV collagenase (Sigma-Aldrich, Milan, Italy) for 12–16~h at 37 °C. The obtained cell suspension was filtered twice using 70 μm nylon meshes, washed, and centrifuged for 5 min at 700 × g. The viability was assessed by Trypan Blue (Sigma-Aldrich, Milan, Italy) test identifying 90% to 95% cell survival. Chondrocytes were recovered, seeded into 10-cm diameter tissue culture plates, and were expanded for 10–12 days in a monolayer incubator with 5% CO_2_ and 90% humidified atmosphere at 37 °C until it reached 80% confluence. For growth and expansion, cells were cultured in Dulbecco’s Modified Eagle Medium (DMEM) (Euroclone, Milan, Italy) with phenol red and L-glutamine, supplemented with 10% fetal bovine serum (FBS) (Euroclone, Milan, Italy), and 200 U/mL penicillin and 200 µg/mL streptomycin (P/S) (Sigma-Aldrich, Milan, Italy). The medium was changed every 2–3 days. The cell morphology was examined daily with an inverted microscope (Olympus IMT-2, Tokyo, Japan), and OA primary chondrocytes at the first passage were employed for the experiments to guarantee their phenotypic stability preserved which can occur when sub-cultured in monolayer [76]. For each single experiment a cell culture from a unique donor was used.

### 4.2. Exposure of Chondrocytes to HP

The HP was generated by a prototype of pressurization system, provided by a hermetically sealed pressure chamber consisting in a stainless-steel cylinder of 400 mm of height and 90 mm of internal diameter [7,15,17]. The chamber was filled with distilled water and was maintained at a constant temperature of 37 °C by a thermostat system. The pressurization was obtained by the hydraulic energy, produced by an electro-power pack, and applied through the transfer accumulator to the water in the chamber. A data processing system, installed in a computer and based on turbo-Pascal language, allowed to preset and modify the pressure inside the chamber for the entire duration of the experiment. 

This system can reach pressure levels ranging between 0 and 240 atm (0–24 MPa), while automatically carrying out the entire program of the filling, thermostatic control steps, and cycles of pressurization of the chamber according to preselected periodic functions. The loading and unloading periods for cyclic pressure, its magnitude, frequency, and duration can be freely selected by the operator. 

OA chondrocytes were seeded in Petri dishes (35 × 10 mm) at a starting density of 1 × 10^5^ cells, until they became 85% confluent, in DMEM supplemented with 10% FBS. Then, the medium was removed, and substituted with DMEM with 0.5% FBS for the treatment procedure. Petri dishes were completely filled with the culture medium and sealed with a special membrane (Surlyn 1801 Bynel CXA 3048 bilayer membrane, Du Pont) after excluding all air to avoid implosions due to the presence of air between the membrane and the medium, suitable for preserving a stable environment. The dishes were placed inside the pressure chamber filled with distilled water at a temperature of 37 °C. The chondrocytes were then pressurized according to sinusoidal waves with a minimum pressure of 1 MPa and a maximum pressure of 5 MPa, frequency of 0.25 Hz, or to static continuous pressure of 10 MPa, for a period of 3~h. Some dishes were maintained in the same culture conditions without receiving any pressurization and were used as controls. Chondrocytes and supernatants at basal conditions and immediately after receiving pressure were collected to perform flow cytometry, quantitative real-time PCR, and immunofluorescence analysis.

### 4.3. RNA Isolation and Quantitative Real-time PCR

Cells were grown and maintained in 6-well dishes at a starting density of 1 × 10^5^ cells/well until they became 85% confluent in DMEM supplemented with 10% FBS, before replacement with 0.5% FBS used for the treatment procedure. Afterwards, cells were collected and total RNA, including miRNA, was extracted using TriPure Isolation Reagent (Euroclone, Milan, Italy) according to the manufacturer’s instructions, and was stored at −80 °C. The concentration, purity, and integrity of RNA were evaluated by measuring the OD at 260 nm and the 260/280 and 260/230 ratios by Nanodrop-1000 (Celbio, Milan, Italy). The quality of RNA was verified by electrophoresis on agarose gel (FlashGel System, Lonza, Rockland, ME, USA). Reverse transcription for target genes was carried out by the QuantiTect Reverse Transcription Kit (Qiagen, Germany), while for miRNA by the cDNA miScript PCR Reverse Transcription (Qiagen, Hilden, Germany), according to the manufacturer’s instructions. 

Target genes and miRNA were examined by real-time PCR using QuantiFast SYBR Green PCR (Qiagen, Hilden, Germany) and miScript SYBR Green (Qiagen, Hilden, Germany) kits, respectively. A list of the used primers is reported in Table 1. 

All qPCR reactions were achieved in glass capillaries by a LightCycler 1.0 (Roche Molecular Biochemicals, Mannheim, Germany) with LightCycler Software Version 3.5. The reaction procedure for miRNA consisted of 95 °C for 15 min for HotStart polymerase activation, followed by 40 cycles of 15 s at 95 °C for denaturation, 30 s at 55 °C for annealing, and 30 s at 70 °C for elongation, according to the protocol. Target gene amplification was performed at 5 s at 95 °C, 40 cycles of 15 s at 95 °C, and 30 s at 60 °C. In the final step of both protocols, the temperature was raised from 60 °C to 95 °C at 0.1 °C/step to plot the melting curve. To analyze the dissociation curves, we visualized the amplicons lengths in agarose gel to confirm the correct amplification of the resulting PCR products.

For the data analysis, the Ct values of each sample and the efficiency of the primer set were calculated through LinReg Software [77] and then converted into relative quantities and normalized according to Pfaffl model [78]. The normalization was performed considering Actin Beta (*ACTB*) for target genes and Small Nucleolar RNA, C/D Box 25 (*SNORD-25*) for miRNA, as the housekeeping genes. These genes were chosen according to geNorm software version 3.5 (Primer design, Southampton University’s School of Medicine, Southampton, UK) [79].

### 4.4. Detection of Apoptosis

The evaluation of apoptotic cells was developed by using Annexin V-FITC and propidium iodide (PI) (ThermoFisher Scientific, Milan, Italy) kit. OA chondrocyte were seeded in 12-well plates (8 × 10^4^ cells/well) for 24~h in DMEM with 10% FBS, before replacement with 0.5% FBS used for the treatment procedure. After that, the cells were washed and harvested by using trypsin, collected into cytometry tubes, and centrifuged at 1500 rpm for 10 min. The supernatant was replaced, and the pellet was resuspended in 100 μL of 1 × Annexin-binding buffer, 5 μL of Alexa Fluor 488 annexin-V conjugated to fluorescein (green fluorescence), and 1 μL of 100 μg/mL PI (red fluorescence) working solution. They were added to 100 μL of cell suspension. Cells were incubated at room temperature for 15 min in the dark. Then, 600 μL of 1 × Annexin-binding buffer were added before the analysis at flow cytometer. A total of 10,000 events (1 × 10^4^ cells per assay) were measured by the instrument. The obtained results were analyzed with Cell Quest software (Version 4.0, Becton Dickinson, San Jose, CA, USA). 

The evaluation of apoptosis was carried out considering staining cells simultaneously with Alexa Fluor 488 annexin-V and PI; this allowed to discriminate intact cells (annexin-V and PI-negative), early apoptosis (annexin-V-positive and PI-negative), and late apoptosis (annexin-V and PI positives) [80]. The results were expressed as a percentage of positive cells to each dye (total apoptosis), and the data were represented as the mean of three independent experiments.

### 4.5. Mitochondrial Superoxide Anion (·O_2_−) Production

OA chondrocyte were seeded in 12 well-plates (8 × 10^4^ cells/well) for 24~h in DMEM with 10% FCS, before replacement with 0.5% FBS used for the treatment procedure. Then, the cells were incubated in Hanks’ Balanced Salt Solution (HBSS) (Euroclone, Milan, Italy) and MitoSOX Red for 15 min at 37 °C in dark, to evaluate mitochondrial superoxide anion (·O_2_−) production. MitoSOX was dissolved in dimethyl sulfoxide (DMSO) (Euroclone, Milan, Italy), at a final concentration of 5 µM. Cells were then harvested by trypsin and collected into cytometry tubes and centrifuged at 1500 rpm for 10 min. Afterwards, cells were dissolved in saline solution before being analyzed by flow cytometry. A density of 1 × 10^4^ cells per assay (a total of 10,000 events) were measured by flow cytometry and data were analyzed with CellQuest software (Version 4.0, Becton Dickinson, San Jose, CA, USA). Results were collected as median of fluorescence (AU) and represented the mean of three independent experiments.

### 4.6. Cell Transfection

Cells were grown and maintained in 6-well dishes at a starting density of 1 × 10^5^ cells/well until they became 85% confluent in DMEM supplemented with 10% FBS, before replacement with 0.5% FBS for 6hr until transfection procedure. Afterwards, chondrocytes were transfected with miR-34a, miR-146a, and miR-181a specific inhibitors (Qiagen, Hilden, Germany), at the concentration of 50 nM, or with the negative controls siRNA (NC) (Qiagen, Hilden, Germany), at the concentration of 5 nM, in serum-free medium for 24~h. Media were removed and the cells were immediately collected or exposed to cycles of low sinusoidal or static continuous HP for a period of 3~h [29,30].

### 4.7. Immunofluorescence Analysis

Human OA chondrocytes were plated in coverslips in 6-well dishes at a starting low density of 4 × 10^4^ cells/chamber, to prevent possible cell overlapping, and re-suspended in 2 mL of culture medium (DMEM + 10% FBS) until 80% of confluence. The medium was replaced with serum-free DMEM, and the cells were processed after 24~h of transfection with miR-34a, miR-146a, and miR-181a specific inhibitors to evaluate the potential activation of the Wnt/β-catenin pathway. Chondrocytes were washed in PBS and then fixed in 4% paraformaldehyde (ThermoFisher Scientific, Milan, Italy) (pH 7.4) for 15 min at room temperature. Afterwards, the cells were permeabilized with a blocking solution (PBS, 1% bovine serum albumin (BSA) (Sigma-Aldrich, Milan, Italy) and 0.2% Triton X-100 (ThermoFisher Scientific, Milan, Italy) for 20 min at room temperature, and then incubated overnight at 4 °C with mouse monoclonal anti-total β-catenin primary antibody (sc-12F7, Santa Cruz Biotechnology, Italy) diluted at 1:250 in PBS, 1% BSA and 0.05% Triton X-100. Three washes in PBS of the coverslips were followed by 1 hr incubation with goat anti-mouse IgG-Texas Red conjugated antibody (Southern Biotechnology, Italy) diluted at 1:100 in PBS, 1% BSA and 0.05% Triton X-100. Finally, the coverslips were washed three times in PBS and submitted to nuclear counterstain by 4,6-diamidino-2-phenylindole (DAPI), and then mounted with Vecta shield (Vector Labs). Fluorescence was examined under a AxioPlan (Zeiss, Oberkochen, Germany) light microscope equipped with epifluorescence at 200× and 400× magnification. The negative controls were obtained by omitting the primary antibody. Immunoreactivity of β-catenin was semi-quantified as the mean densitometric area of protein signal into the nucleus and into the cytoplasm, by AxioVision 4.6 software measure program (Zeiss, Oberkochen, Germany) [81]. At least 100 chondrocytes from each group were evaluated.

### 4.8. Statistical Analysis

Three independent experiments were carried out and the results were expressed as the mean ± standard deviation (SD) of triplicate values for each experiment. Data normal distribution was evaluated by Shapiro–Wilk, D’Agostino and Pearson, and Kolmogorov–Smirnov tests. Flow cytometry results were analyzed by ANOVA with Bonferroni post-hoc test. Quantitative real-time PCR, immunofluorescence and Western blot data were evaluated by one-way ANOVA with a Tukey’s post-hoc test using 2^−ΔΔCT^ values for each sample. All analyses were performed through the SAS System (SAS Institute Inc., Cary, NC, USA) and GraphPad Prism 6.1. A *p*-value <0.05 was defined as statistically significant.

## 5. Conclusions

The results of the present study add new information about the role of HP in the regulation of cartilage metabolism and homeostasis, and demonstrate that the response of the cell depends on the magnitude and frequency of HP. 

First of all, we showed that a low cyclic HP (1–5 MPa), approximating the physiological condition of human joints, was able to inhibit the expression levels of matrix degrading enzymes and to restore *Col2a1* mRNA in human OA chondrocytes; besides, this pressure reduced apoptosis signaling and up-regulated the anti-apoptotic marker *BCL2*, and reduced ROS production and antioxidant genes expression (*SOD-2, NRF2*). Furthermore, low HP down-regulated the gene expression of *miR-34a*, *miR-146a*, and *miR-181a*, known to be involved in OA pathogenesis. On the other hand, opposite and detrimental effects were observed when the chondrocytes were exposed to static continuous HP of 10 MPa. Additionally, the transient inhibition of miR-34a, miR-146a, and miR-181a confirmed their role in the regulation of cartilage turnover, apoptosis and oxidative stress processes, enhancing the positive effects of low cyclic pressure, whereas limiting the negative activity of overloading HP. Moreover, the silencing of these miRNA influenced β-catenin expression regulated by HP.

Taken together, our results demonstrate how a specific application and intensity of HP stimulation could differently influence cartilage integrity, suggesting that a dynamic physiological loading seems to have protective effects on chondrocyte metabolism, while a static continuous pressure activates damaging processes for the cell. Besides, we can suppose that miRNA may represent potential mediators by which mechanical loading regulates cartilage turnover, apoptosis signaling and oxidative stress balance, probably via Wnt/β-catenin pathway. 

However, the results of this paper are preliminary and additional experiments are required to support our findings, for instance, to deeper investigate the role of HP on oxidant/antioxidant balance, and to better elucidate the involvement of miRNA in mediating HP effects on oxidative stress. In particular, considering that NRF2/KEAP1 pathway is a master regulator of cell protection mechanism against ROS sources, the use of NRF2 activators or inhibitors could be a promising approach to further investigate oxidative stress regulation by HP. In addition, the protein levels of β-catenin at western blot analysis should be detected to support IF results, and the use of β-catenin specific inhibitor is necessary to confirm the involvement of the pathway in HP-mediated oxidative stress regulation. An evaluation of Wnt/β-catenin after miR-146a and miR-181a is recommended to attest the role of these miRNA in mediating HP effect on protein expression. In addition, a more deeper investigation on NRF2 and β-catenin by using electrophoretic mobility shift assay or luciferase assay could contribute to better examine the activities of these transcriptional factors. An IF analysis by using different antibodies able to distinguish between total and phosphor-β-catenin should be useful to understand which form of the protein is interested in mediating HP effects. Additional experiments on healthy primary cells are also suggested to prove the involvement of mechanical loading in influencing chondrocytes metabolism in the pathogenesis of OA. Finally, further researches are required to identify the ideal mechanical loading contributing to prevent the development and the progression of OA, and to translate this information for the therapeutic program of OA patients.

## Figures and Tables

**Table 1:** Primers used for qRT-qPCR.

Target genes	Cat. No. (Qiagen)
*MMP-13*	QT00001764
*ADAMTS-5*	QT00011088
*Col2a1*	QT00049518
*BCL2*	QT00000721
*SOD-2*	QT01008693
*NRF2*	QT00027384
*ACTB*	QT00095431
**miRNA Genes**	**Cat. No. (Qiagen)**
*miR-34a*	MS00003318
*miR-146a*	MS00003535
*miR-181a*	MS00006692
*SNORD-25*	MS00014007

Abbreviations: *MMP-13* = metalloproteinase 13; *ADAMTS-5* = metalloproteinase with thrombospondin motif; *Col2a1* = type II collagen; *BCL2* = B-cell lymphoma 2; *SOD-2* = superoxide dismutase 2; *NRF2* = nuclear factor erythroid 2 like 2; *ACTB* = Actin Beta; *miRNA* = microRNA; *SNORD-25* = Small Nucleolar RNA, C/D Box 25.

## Supplementary Materials

The following are available online at https://www.mdpi.com/1422-0067/21/10/3653/s1, Figure S1. Apoptosis detection analyzed at flow cytometry and measured with Annexin Alexa fluor 488 assay. Figure S2. MitoSOX-based flow cytometric detection of mitochondrial superoxide anion production at basal condition (Basal, cells without treatment).
