# Supplementary figures and images for: Hydrostatic Pressure Regulates Oxidative Stress through microRNA in Human Osteoarthritic Chondrocytes
Source: Int J Mol Sci. 2020 May 21;21(10):3653. doi: 10.3390/ijms21103653 (PMC7279254; doi:10.3390/ijms21103653)

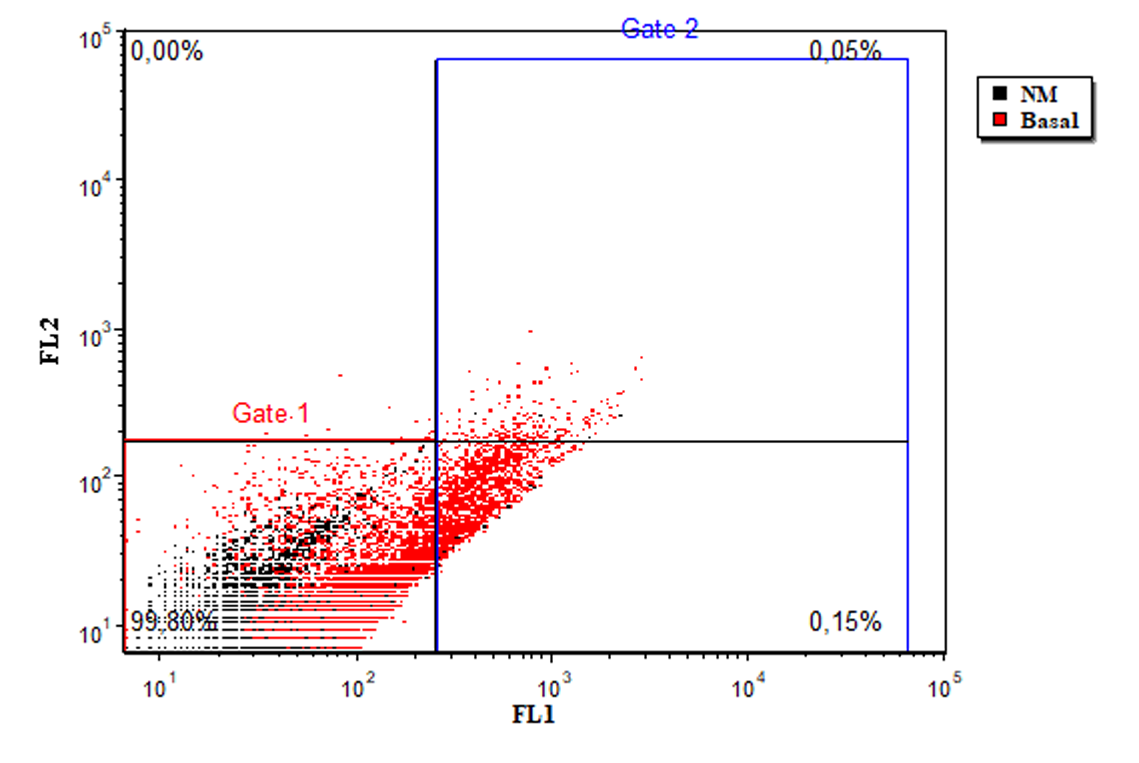

Supplement: Supplementary file 1 [file ijms-21-03653-s001.zip › Figure S1.tif]

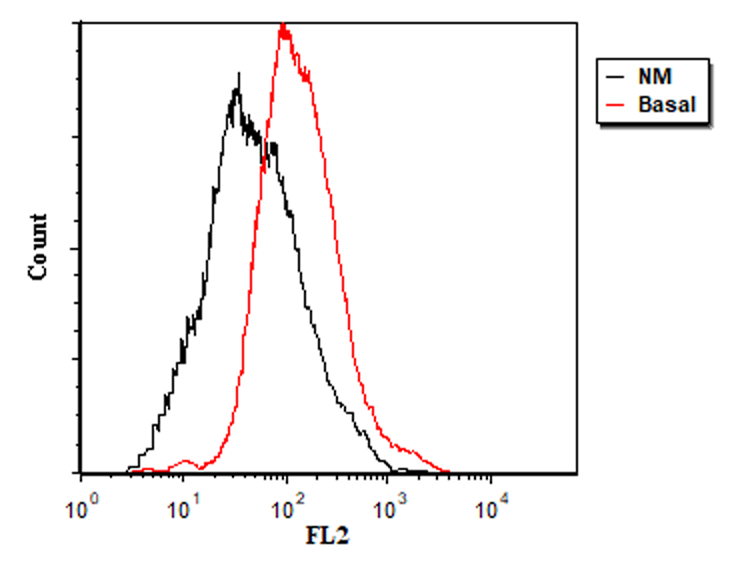

Supplement: Supplementary file 1 [file ijms-21-03653-s001.zip › Figure S2.tif]
